# Supplementary material for: Archaeal G-quadruplexes: a novel model for understanding unusual DNA/RNA structures across the tree of life
Source: Nucleic Acids Res. 2026 Feb 5;54(4):gkag067. doi: 10.1093/nar/gkag067 (PMC12873603; doi:10.1093/nar/gkag067)

Supplemental Figure 1

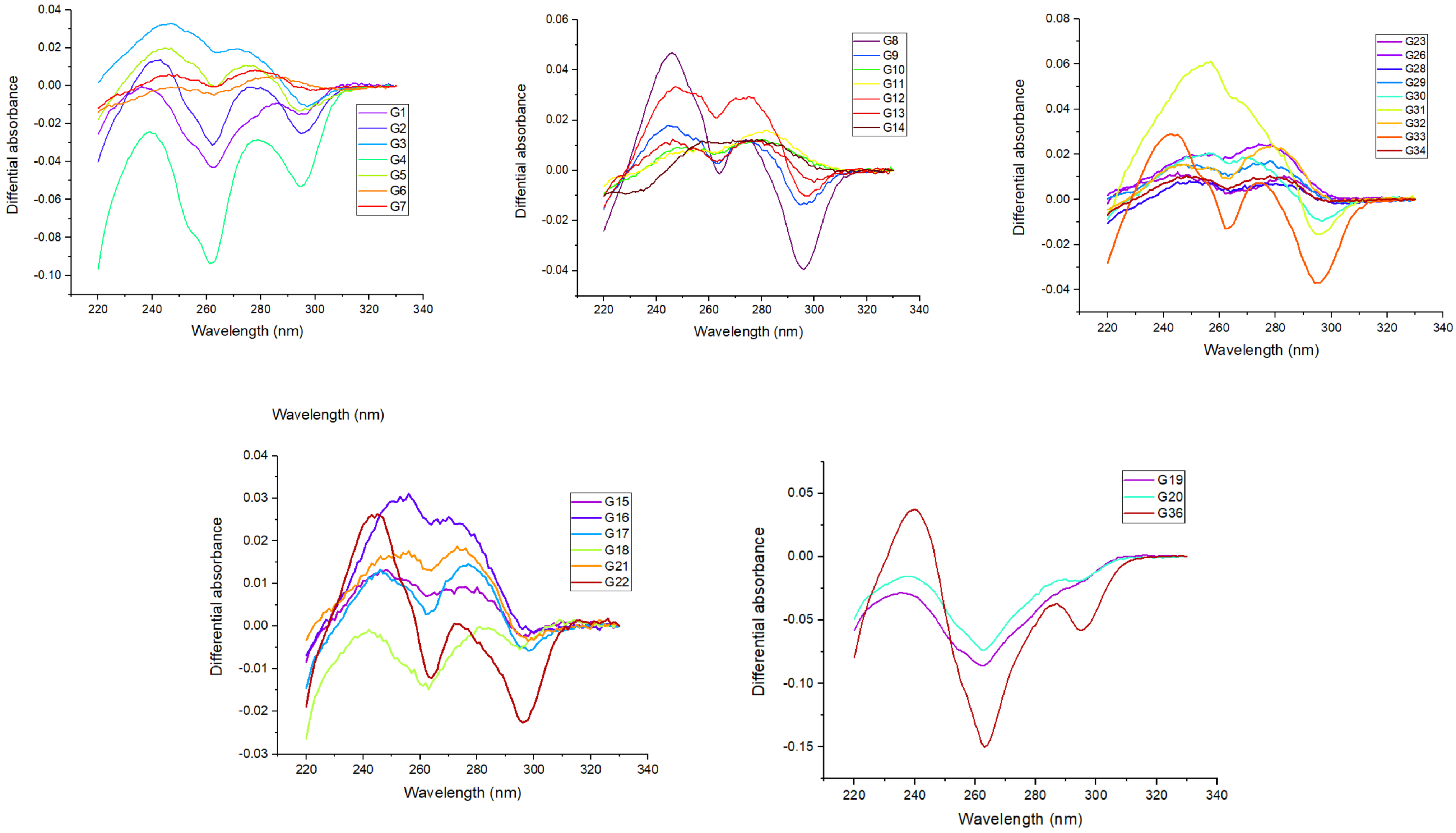

Supplemental Figure 2

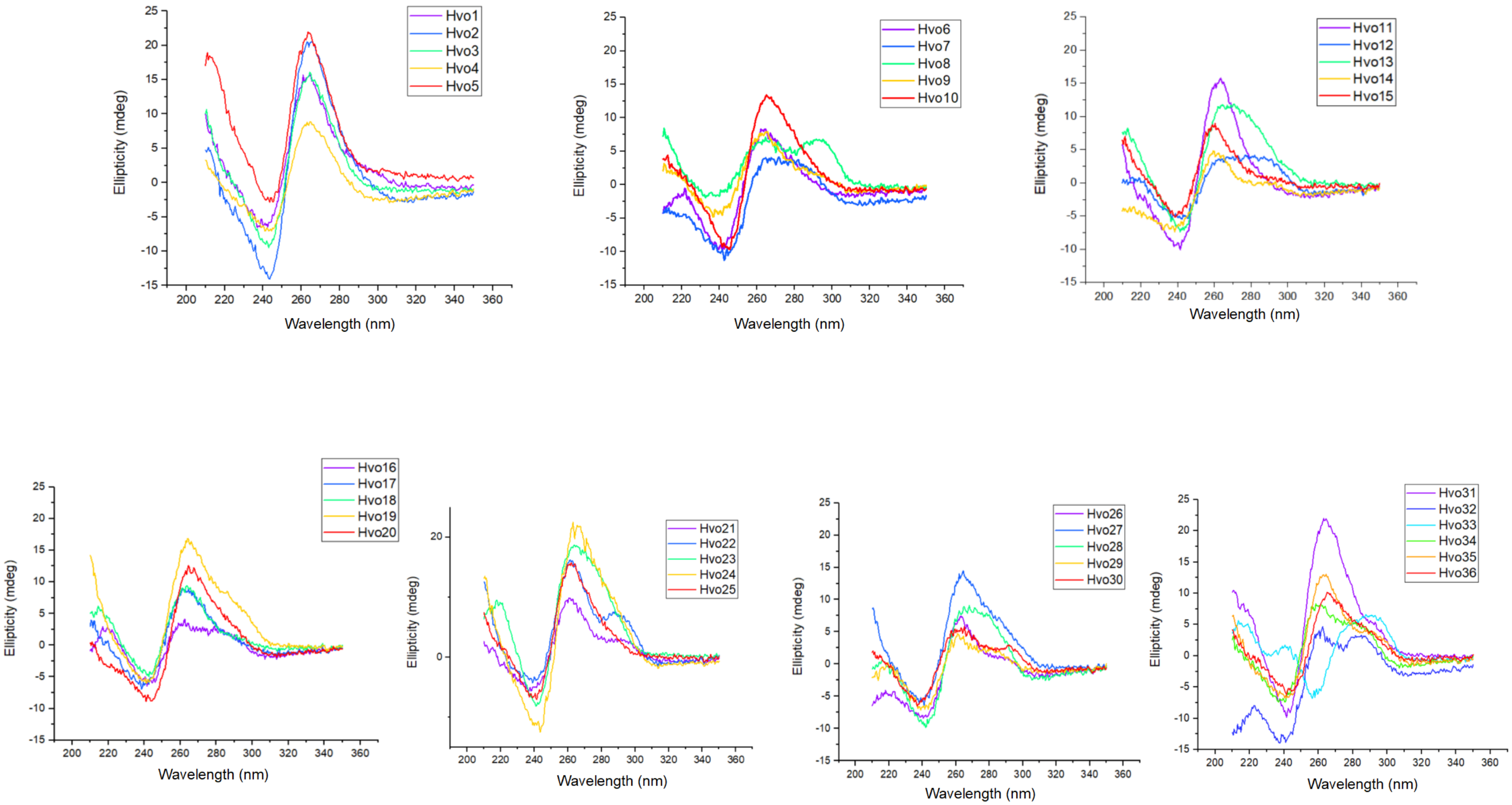

Supplemental Figure 3

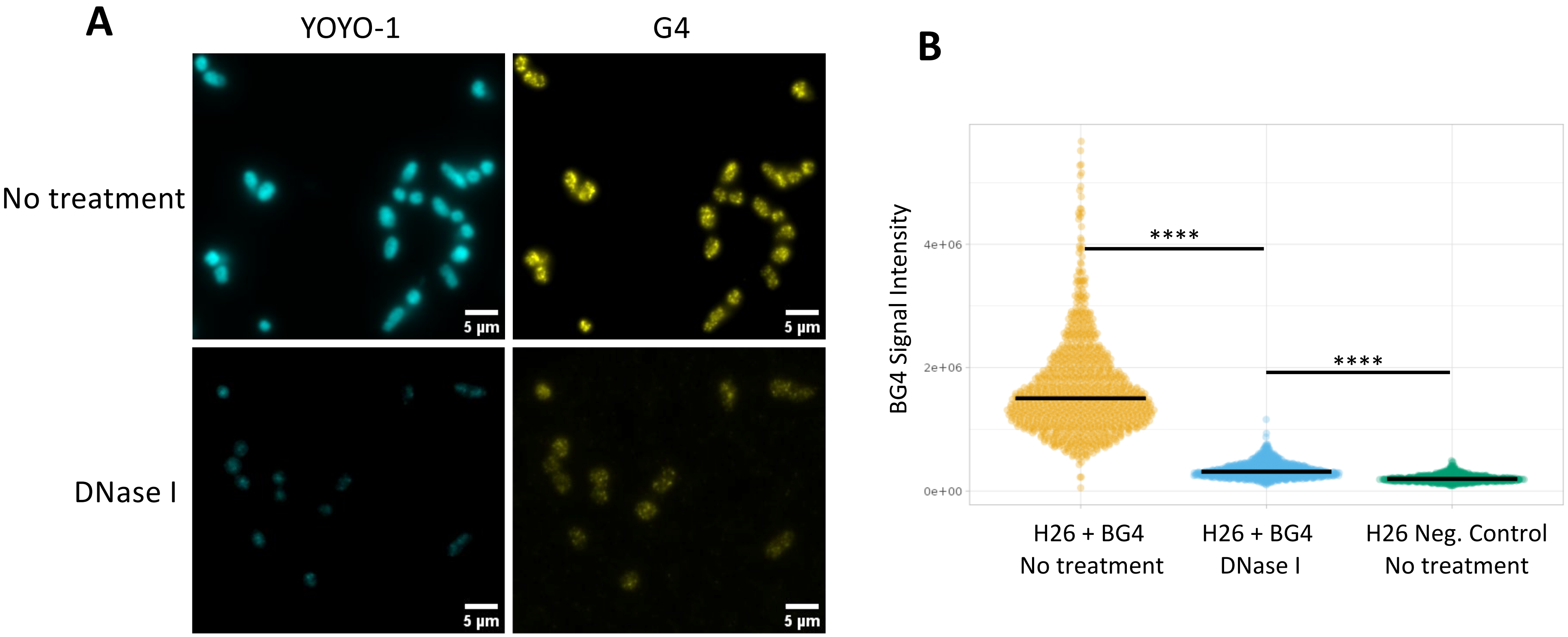

Supplemental Figure 4

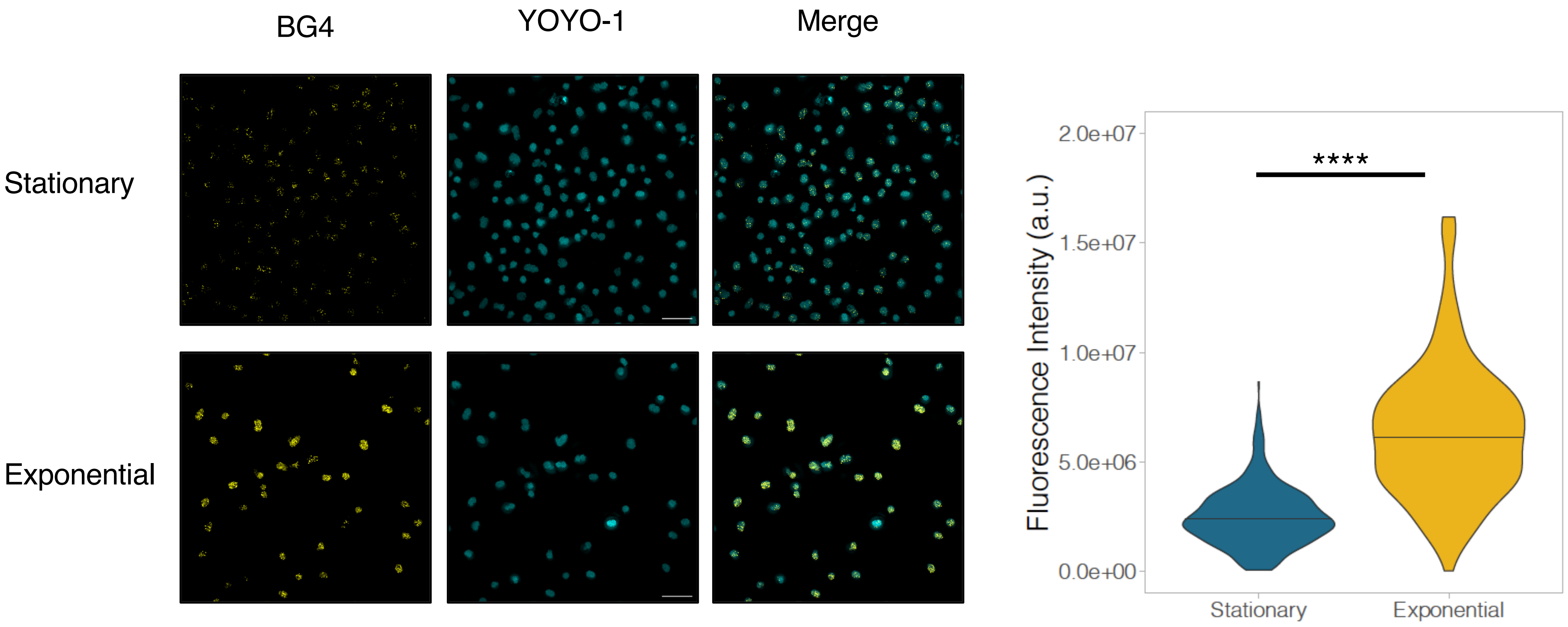

Supplemental Figure 5

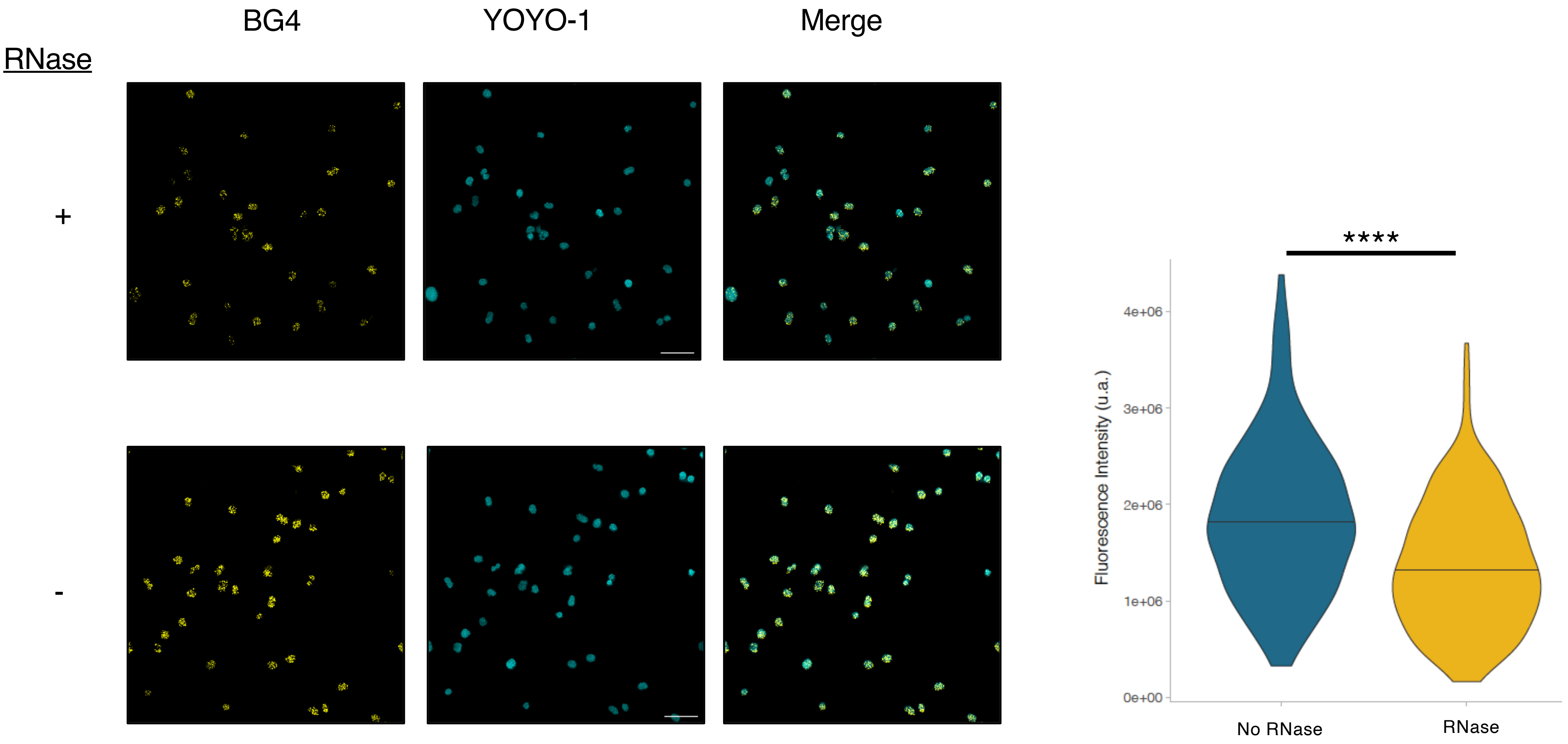

Supplemental Figure 6

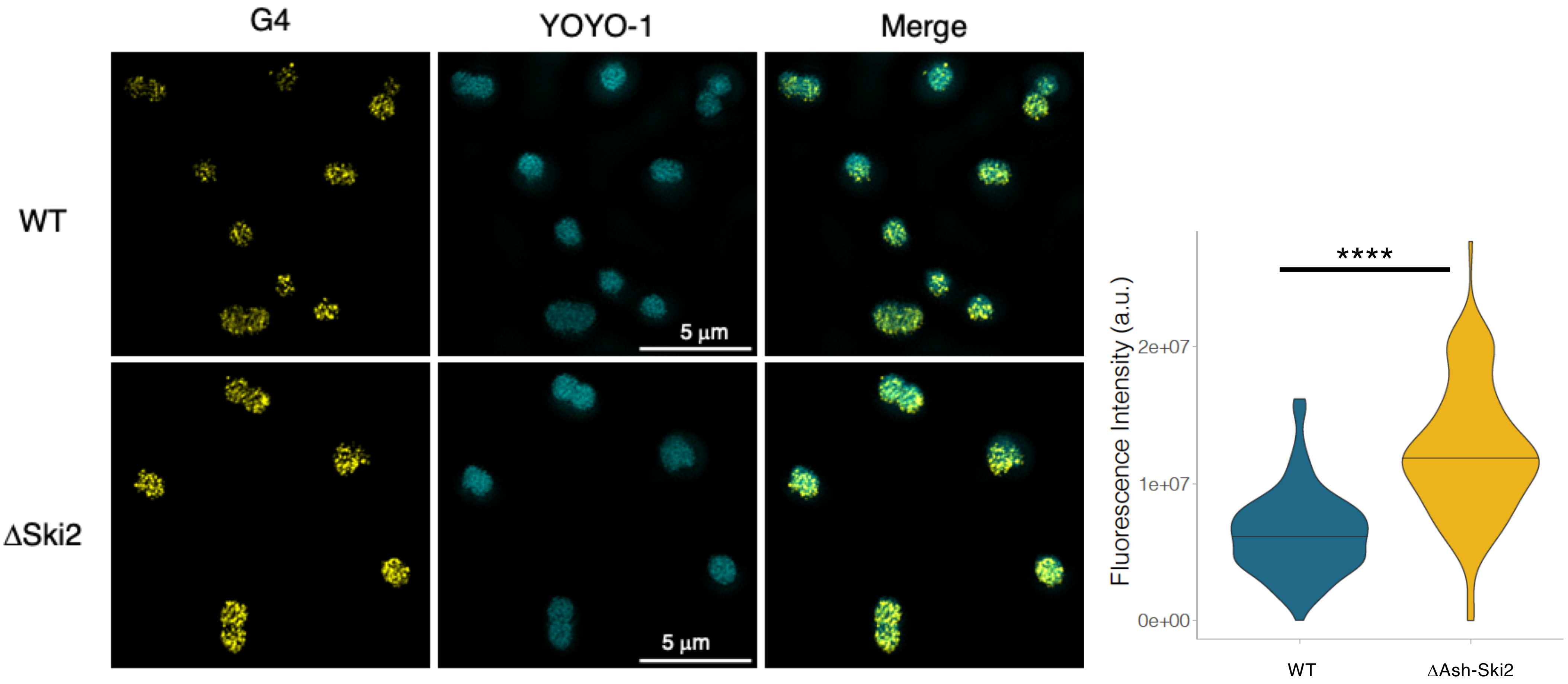

Supplement: gkag067_Supplemental_Files [file gkag067_supplemental_files.zip › Figures S1-S6.pdf]
