# Supplementary material for: Archaeal G-quadruplexes: a novel model for understanding unusual DNA/RNA structures across the tree of life
Source: Nucleic Acids Res. 2026 Feb 5;54(4):gkag067. doi: 10.1093/nar/gkag067 (PMC12873603; doi:10.1093/nar/gkag067)
Supplement: gkag067_Supplemental_Files [file gkag067_supplemental_files.zip › Supplemental Tables and Legends NAR -revised.docx]

**Supplemental Table 1.** DNA oligonucleotides from *H. volcanii* used to study G4 folding.

All sequences are provided in the 5’ 🡪 3’ direction.

**Supplemental Table 2**. DNA oligonucleotides, positive and negative controls for G4 folding, used in the present study. All sequences are provided in the 5’ 🡪 3’

**Supplemental Table 3.** Oligonucleotides used in the present study to generate Rad3a/b knock-out strains by the SLIC method and the Dna2 knock-out strain. All sequences are provided in the 5’ 🡪 3’ (US : Upstream and DS : Downstream)

**Supplemental Table S4 : Table summarizing the conclusions for all experimentally tested sequences.**

| **Name** | **FRET MC** | **ThT** | **NMM** | **IDS** | **CD** | **Conclusion** |
| --- | --- | --- | --- | --- | --- | --- |
| **Hvo-G1** |  |  |  |  |  |  |
| **Hvo-G2** |  |  |  |  |  |  |
| **Hvo-G3** |  |  |  |  |  |  |
| **Hvo-G4** |  |  |  |  |  |  |
| **Hvo-G5** |  |  |  |  |  |  |
| **Hvo-G6** |  |  |  |  |  |  |
| **Hvo-G7** |  |  |  |  |  |  |
| ***Hvo-G8*** |  |  |  |  |  |  |
| ***Hvo-G9*** |  |  |  |  |  |  |
| **Hvo-G10** |  |  |  |  |  |  |
| **Hvo-G11** |  |  |  |  |  |  |
| **Hvo-G12** |  |  |  |  |  |  |
| ***Hvo-G13*** |  |  |  |  |  |  |
| **Hvo-G14** |  |  |  |  |  |  |
| ***Hvo-G15*** |  |  |  |  |  |  |
| ***Hvo-G16*** |  |  |  |  |  |  |
| ***Hvo-G17*** |  |  |  |  |  |  |
| ***Hvo-G18*** |  |  |  |  |  |  |
| ***Hvo-G19*** |  |  |  |  |  |  |
| ***Hvo-G20*** |  |  |  |  |  |  |
| ***Hvo-G21*** |  |  |  |  |  |  |
| ***Hvo-G22*** |  |  |  |  |  |  |
| ***Hvo-G23*** |  |  |  |  |  |  |
| ***Hvo-G24*** |  |  |  |  |  |  |
| ***Hvo-G25*** |  |  |  |  |  |  |
| ***Hvo-G26*** |  |  |  |  |  |  |
| ***Hvo-G27*** |  |  |  |  |  |  |
| ***Hvo-G28*** |  |  |  |  |  |  |
| ***Hvo-G29*** |  |  |  |  |  |  |
| ***Hvo-G30*** |  |  |  |  |  |  |
| ***Hvo-G31*** |  |  |  |  |  |  |
| ***Hvo-G32*** |  |  |  |  |  |  |
| ***Hvo-G33*** |  |  |  |  |  |  |
| ***Hvo-G34*** |  |  |  |  |  |  |
| ***Hvo-G35*** |  |  |  |  |  |  |
| ***Hvo-G36*** |  |  |  |  |  |  |

|  | G4 forming |
| --- | --- |
|  | Likely to form G4 (unstable or at low propensity) |
|  | Unlikely to form G4 |
|  | Non determined |

**Supplemental figure legends**

**Supplemental Fig. 1.** Isothermal difference spectra (IDS) of the *H. volcanii* sequences.

**Supplemental Fig. 2.** Circular dichroism spectra of the *H. volcanii* sequences.

**Supplemental Fig. 3.** Immunofluorescence labelling and quantification of G4s in fixed *T. barophilus* cells in exponential and stationary phase. Statistical analyses were performed using a Mann-Whitney test. **** p > 0.0001. Bar, 5 μm.

**Supplemental Fig. 4.** Immunofluorescence labelling and quantification of G4s in fixed *T. barophilus* cells in exponential phase treated with RNase A. Statistical analyses were performed using a Mann-Whitney test. **** p > 0.0001. Bar, 5 μm.

**Supplemental Fig. 5.** Immunofluorescence labelling of G4s in WT or ΔSki2 *T. barophilus* exponential phase cells. Bar, 5 μm.
